# Supplementary material for: Modeling glioblastoma heterogeneity as a dynamic network of cell states
Source: Mol Syst Biol. 2021 Sep 16;17(9):e10105. doi: 10.15252/msb.202010105 (PMC8444284; doi:10.15252/msb.202010105)
Supplement: Supplementary file 6 — Source Data for Figure 5 [file MSB-17-e10105-s004.zip › Figure5A_sourcedata/GSEA_3017/nosigdb_stateF.GseaPreranked.1622795015644/RICHARDS_NOWAKOWSKI_SCIENCE_2017_MGE_IPC2_UPREG.html]

Details for gene set RICHARDS\_NOWAKOWSKI\_SCIENCE\_2017\_MGE\_IPC2\_UPREG[GSEA]

|  || Dataset | state33017 |
| Phenotype | NoPhenotypeAvailable |
| Upregulated in class | na\_neg |
| GeneSet | RICHARDS\_NOWAKOWSKI\_SCIENCE\_2017\_MGE\_IPC2\_UPREG |
| Enrichment Score (ES) | -0.74576473 |
| Normalized Enrichment Score (NES) | -5.796683 |
| Nominal p-value | 0.0 |
| FDR q-value | 0.0 |
| FWER p-Value | 0.0 |
Table: GSEA Results Summary

  

Fig 1: Enrichment plot: RICHARDS\_NOWAKOWSKI\_SCIENCE\_2017\_MGE\_IPC2\_UPREG      
 Profile of the Running ES Score & Positions of GeneSet Members on the Rank Ordered List

  

| PROBE | GENE SYMBOL | GENE\_TITLE | RANK IN GENE LIST | RANK METRIC SCORE | RUNNING ES | CORE ENRICHMENT || 1 | RPS10 |  |  | 119 | 0.717 | -0.0571 | No |
| 2 | DDB2 |  |  | 143 | 0.682 | -0.0654 | No |
| 3 | KAT2B |  |  | 193 | 0.594 | -0.0874 | No |
| 4 | HERPUD1 |  |  | 197 | 0.590 | -0.0859 | No |
| 5 | PIK3R3 |  |  | 251 | 0.553 | -0.1101 | No |
| 6 | FBXW7 |  |  | 272 | 0.537 | -0.1176 | No |
| 7 | GPC6 |  |  | 332 | 0.495 | -0.1452 | No |
| 8 | DCLK2 |  |  | 340 | 0.492 | -0.1463 | No |
| 9 | ABAT |  |  | 362 | 0.483 | -0.1545 | No |
| 10 | RAP2B |  |  | 372 | 0.480 | -0.1567 | No |
| 11 | KITLG |  |  | 401 | 0.470 | -0.1686 | No |
| 12 | TMSB4X |  |  | 590 | 0.401 | -0.2626 | No |
| 13 | GRIK3 |  |  | 598 | 0.399 | -0.2641 | No |
| 14 | KCNJ2 |  |  | 607 | 0.396 | -0.2662 | No |
| 15 | LSAMP |  |  | 611 | 0.394 | -0.2657 | No |
| 16 | PFN2 |  |  | 724 | 0.365 | -0.3211 | No |
| 17 | MSI2 |  |  | 780 | 0.352 | -0.3474 | No |
| 18 | RAB3IP |  |  | 797 | 0.348 | -0.3538 | No |
| 19 | ARHGEF26 |  |  | 885 | 0.328 | -0.3966 | No |
| 20 | DST |  |  | 896 | 0.327 | -0.4000 | No |
| 21 | FAT1 |  |  | 968 | 0.315 | -0.4347 | No |
| 22 | SPECC1 |  |  | 1089 | 0.294 | -0.4945 | No |
| 23 | ZBTB20 |  |  | 1181 | 0.281 | -0.5396 | No |
| 24 | PTPRE |  |  | 1243 | 0.275 | -0.5694 | No |
| 25 | ST8SIA5 |  |  | 1277 | 0.271 | -0.5848 | No |
| 26 | ELAVL4 |  |  | 1358 | 0.261 | -0.6244 | No |
| 27 | GAPDH |  |  | 1361 | 0.261 | -0.6241 | No |
| 28 | MEIS1 |  |  | 1407 | 0.256 | -0.6458 | No |
| 29 | EAF1 |  |  | 1440 | 0.254 | -0.6608 | No |
| 30 | FANCL |  |  | 1442 | 0.253 | -0.6600 | No |
| 31 | DNA2 |  |  | 1479 | -0.252 | -0.6771 | No |
| 32 | DCLRE1C |  |  | 1493 | -0.257 | -0.6825 | No |
| 33 | NCAPH2 |  |  | 1496 | -0.258 | -0.6822 | No |
| 34 | HNRNPA2B1 |  |  | 1503 | -0.261 | -0.6839 | No |
| 35 | BLM |  |  | 1507 | -0.262 | -0.6841 | No |
| 36 | BRD8 |  |  | 1508 | -0.263 | -0.6827 | No |
| 37 | RBBP8 |  |  | 1518 | -0.265 | -0.6860 | No |
| 38 | MAD2L2 |  |  | 1539 | -0.269 | -0.6948 | No |
| 39 | ZNF43 |  |  | 1544 | -0.270 | -0.6955 | No |
| 40 | BAZ1A |  |  | 1547 | -0.271 | -0.6951 | No |
| 41 | NUP62 |  |  | 1562 | -0.275 | -0.7008 | No |
| 42 | PSIP1 |  |  | 1564 | -0.275 | -0.6999 | No |
| 43 | WDHD1 |  |  | 1569 | -0.277 | -0.7005 | No |
| 44 | IQGAP3 |  |  | 1584 | -0.282 | -0.7062 | No |
| 45 | ZNF704 |  |  | 1588 | -0.284 | -0.7063 | No |
| 46 | RCC1 |  |  | 1604 | -0.286 | -0.7125 | No |
| 47 | CCDC150 |  |  | 1605 | -0.286 | -0.7110 | No |
| 48 | CNTRL |  |  | 1610 | -0.288 | -0.7116 | No |
| 49 | HP1BP3 |  |  | 1618 | -0.290 | -0.7137 | No |
| 50 | RSRC1 |  |  | 1633 | -0.294 | -0.7193 | No |
| 51 | GAS2L3 |  |  | 1640 | -0.297 | -0.7209 | No |
| 52 | APOLD1 |  |  | 1648 | -0.299 | -0.7229 | No |
| 53 | ILDR2 |  |  | 1650 | -0.300 | -0.7219 | No |
| 54 | CEP57 |  |  | 1676 | -0.309 | -0.7331 | No |
| 55 | RDX |  |  | 1678 | -0.310 | -0.7320 | No |
| 56 | NUP107 |  |  | 1683 | -0.313 | -0.7324 | No |
| 57 | G2E3 |  |  | 1687 | -0.315 | -0.7323 | No |
| 58 | MGME1 |  |  | 1697 | -0.317 | -0.7353 | No |
| 59 | PRIM1 |  |  | 1703 | -0.320 | -0.7362 | No |
| 60 | H1FX |  |  | 1705 | -0.321 | -0.7351 | No |
| 61 | HAUS1 |  |  | 1708 | -0.322 | -0.7345 | No |
| 62 | CENPQ |  |  | 1711 | -0.322 | -0.7338 | No |
| 63 | CDC25C |  |  | 1724 | -0.327 | -0.7383 | No |
| 64 | RALY |  |  | 1729 | -0.330 | -0.7386 | No |
| 65 | BRD7 |  |  | 1730 | -0.330 | -0.7369 | No |
| 66 | DSEL |  |  | 1735 | -0.333 | -0.7373 | No |
| 67 | PPP2R3C |  |  | 1736 | -0.335 | -0.7355 | No |
| 68 | MCM3 |  |  | 1738 | -0.335 | -0.7343 | No |
| 69 | PLK4 |  |  | 1740 | -0.336 | -0.7331 | No |
| 70 | RMI1 |  |  | 1743 | -0.337 | -0.7324 | No |
| 71 | STIL |  |  | 1744 | -0.337 | -0.7307 | No |
| 72 | EXOSC9 |  |  | 1746 | -0.338 | -0.7294 | No |
| 73 | PAICS |  |  | 1757 | -0.342 | -0.7328 | No |
| 74 | ZNF90 |  |  | 1763 | -0.345 | -0.7336 | No |
| 75 | TOPBP1 |  |  | 1781 | -0.354 | -0.7405 | No |
| 76 | WDR76 |  |  | 1783 | -0.355 | -0.7391 | No |
| 77 | SRPK1 |  |  | 1788 | -0.356 | -0.7394 | No |
| 78 | BRIP1 |  |  | 1790 | -0.357 | -0.7380 | No |
| 79 | KIF20A |  |  | 1792 | -0.358 | -0.7367 | No |
| 80 | ILF2 |  |  | 1795 | -0.360 | -0.7359 | No |
| 81 | LIG1 |  |  | 1797 | -0.361 | -0.7345 | No |
| 82 | CENPL |  |  | 1805 | -0.362 | -0.7362 | No |
| 83 | NEK2 |  |  | 1815 | -0.371 | -0.7389 | No |
| 84 | SAP30 |  |  | 1826 | -0.378 | -0.7421 | No |
| 85 | C18orf54 |  |  | 1830 | -0.380 | -0.7417 | No |
| 86 | BARD1 |  |  | 1839 | -0.384 | -0.7438 | Yes |
| 87 | ZNF519 |  |  | 1841 | -0.385 | -0.7423 | Yes |
| 88 | MASTL |  |  | 1843 | -0.385 | -0.7409 | Yes |
| 89 | MMS22L |  |  | 1846 | -0.387 | -0.7399 | Yes |
| 90 | SMCHD1 |  |  | 1847 | -0.387 | -0.7379 | Yes |
| 91 | SRSF10 |  |  | 1851 | -0.390 | -0.7374 | Yes |
| 92 | CEP135 |  |  | 1855 | -0.393 | -0.7369 | Yes |
| 93 | KIF18B |  |  | 1860 | -0.393 | -0.7370 | Yes |
| 94 | FANCB |  |  | 1862 | -0.396 | -0.7354 | Yes |
| 95 | E2F8 |  |  | 1864 | -0.396 | -0.7339 | Yes |
| 96 | EXOSC8 |  |  | 1865 | -0.397 | -0.7319 | Yes |
| 97 | HIST1H3D |  |  | 1867 | -0.398 | -0.7303 | Yes |
| 98 | CHD7 |  |  | 1874 | -0.400 | -0.7313 | Yes |
| 99 | CENPO |  |  | 1884 | -0.410 | -0.7338 | Yes |
| 100 | RFC4 |  |  | 1887 | -0.413 | -0.7327 | Yes |
| 101 | SEPT10 |  |  | 1888 | -0.414 | -0.7306 | Yes |
| 102 | POLQ |  |  | 1891 | -0.417 | -0.7295 | Yes |
| 103 | YBX1 |  |  | 1892 | -0.417 | -0.7273 | Yes |
| 104 | SAE1 |  |  | 1893 | -0.417 | -0.7252 | Yes |
| 105 | HDAC2 |  |  | 1896 | -0.418 | -0.7241 | Yes |
| 106 | RAD18 |  |  | 1898 | -0.419 | -0.7224 | Yes |
| 107 | E2F3 |  |  | 1907 | -0.429 | -0.7243 | Yes |
| 108 | BAZ1B |  |  | 1910 | -0.429 | -0.7231 | Yes |
| 109 | TTF1 |  |  | 1917 | -0.436 | -0.7239 | Yes |
| 110 | POLD3 |  |  | 1918 | -0.436 | -0.7217 | Yes |
| 111 | UHRF1 |  |  | 1921 | -0.438 | -0.7205 | Yes |
| 112 | DIAPH3 |  |  | 1924 | -0.439 | -0.7192 | Yes |
| 113 | HMGXB4 |  |  | 1928 | -0.441 | -0.7185 | Yes |
| 114 | GINS2 |  |  | 1932 | -0.445 | -0.7177 | Yes |
| 115 | LINC01224 |  |  | 1934 | -0.446 | -0.7160 | Yes |
| 116 | HNRNPDL |  |  | 1940 | -0.448 | -0.7162 | Yes |
| 117 | TTF2 |  |  | 1942 | -0.449 | -0.7144 | Yes |
| 118 | STAG1 |  |  | 1943 | -0.450 | -0.7121 | Yes |
| 119 | CCNF |  |  | 1947 | -0.451 | -0.7113 | Yes |
| 120 | CLIC1 |  |  | 1949 | -0.454 | -0.7095 | Yes |
| 121 | RFC2 |  |  | 1954 | -0.457 | -0.7092 | Yes |
| 122 | DDX39A |  |  | 1957 | -0.458 | -0.7079 | Yes |
| 123 | AGO2 |  |  | 1966 | -0.462 | -0.7096 | Yes |
| 124 | DNMT1 |  |  | 1969 | -0.465 | -0.7082 | Yes |
| 125 | CIT |  |  | 1978 | -0.474 | -0.7098 | Yes |
| 126 | HAUS6 |  |  | 1984 | -0.478 | -0.7099 | Yes |
| 127 | SACS |  |  | 1987 | -0.480 | -0.7085 | Yes |
| 128 | NASP |  |  | 1988 | -0.481 | -0.7060 | Yes |
| 129 | TIMELESS |  |  | 1989 | -0.482 | -0.7035 | Yes |
| 130 | MCM4 |  |  | 1993 | -0.487 | -0.7026 | Yes |
| 131 | SKA1 |  |  | 1996 | -0.490 | -0.7011 | Yes |
| 132 | SPAG5 |  |  | 1997 | -0.490 | -0.6986 | Yes |
| 133 | TRA2B |  |  | 1998 | -0.490 | -0.6960 | Yes |
| 134 | PSMC3 |  |  | 1999 | -0.492 | -0.6935 | Yes |
| 135 | ARL6IP1 |  |  | 2001 | -0.494 | -0.6915 | Yes |
| 136 | FAM111A |  |  | 2002 | -0.497 | -0.6889 | Yes |
| 137 | TK1 |  |  | 2003 | -0.497 | -0.6864 | Yes |
| 138 | TUBB6 |  |  | 2006 | -0.500 | -0.6848 | Yes |
| 139 | XPO1 |  |  | 2007 | -0.500 | -0.6822 | Yes |
| 140 | CTCF |  |  | 2010 | -0.502 | -0.6807 | Yes |
| 141 | CDK5RAP2 |  |  | 2012 | -0.503 | -0.6786 | Yes |
| 142 | CHEK1 |  |  | 2013 | -0.504 | -0.6760 | Yes |
| 143 | HIRIP3 |  |  | 2017 | -0.508 | -0.6749 | Yes |
| 144 | FEN1 |  |  | 2018 | -0.511 | -0.6723 | Yes |
| 145 | SMC1A |  |  | 2021 | -0.513 | -0.6707 | Yes |
| 146 | RRM1 |  |  | 2023 | -0.516 | -0.6686 | Yes |
| 147 | HAUS8 |  |  | 2026 | -0.517 | -0.6669 | Yes |
| 148 | PARPBP |  |  | 2030 | -0.520 | -0.6658 | Yes |
| 149 | DEPDC1B |  |  | 2037 | -0.527 | -0.6661 | Yes |
| 150 | HELLS |  |  | 2038 | -0.528 | -0.6634 | Yes |
| 151 | TPM4 |  |  | 2039 | -0.528 | -0.6607 | Yes |
| 152 | CDCA3 |  |  | 2041 | -0.529 | -0.6585 | Yes |
| 153 | TROAP |  |  | 2046 | -0.533 | -0.6578 | Yes |
| 154 | TTK |  |  | 2047 | -0.533 | -0.6551 | Yes |
| 155 | NCAPD3 |  |  | 2051 | -0.537 | -0.6538 | Yes |
| 156 | KIF22 |  |  | 2053 | -0.541 | -0.6516 | Yes |
| 157 | CENPJ |  |  | 2054 | -0.544 | -0.6488 | Yes |
| 158 | STMN1 |  |  | 2063 | -0.567 | -0.6499 | Yes |
| 159 | PTMA |  |  | 2065 | -0.570 | -0.6475 | Yes |
| 160 | DTL |  |  | 2066 | -0.573 | -0.6446 | Yes |
| 161 | FANCD2 |  |  | 2068 | -0.577 | -0.6421 | Yes |
| 162 | ZWINT |  |  | 2069 | -0.577 | -0.6391 | Yes |
| 163 | CEP152 |  |  | 2070 | -0.578 | -0.6362 | Yes |
| 164 | RTKN2 |  |  | 2071 | -0.579 | -0.6332 | Yes |
| 165 | RACGAP1 |  |  | 2072 | -0.579 | -0.6302 | Yes |
| 166 | MND1 |  |  | 2074 | -0.586 | -0.6277 | Yes |
| 167 | SKA3 |  |  | 2077 | -0.590 | -0.6257 | Yes |
| 168 | BUB3 |  |  | 2078 | -0.596 | -0.6226 | Yes |
| 169 | AURKB |  |  | 2079 | -0.598 | -0.6195 | Yes |
| 170 | SRSF3 |  |  | 2081 | -0.606 | -0.6169 | Yes |
| 171 | CENPK |  |  | 2082 | -0.607 | -0.6138 | Yes |
| 172 | HNRNPR |  |  | 2083 | -0.612 | -0.6107 | Yes |
| 173 | USP1 |  |  | 2084 | -0.612 | -0.6075 | Yes |
| 174 | CKAP5 |  |  | 2086 | -0.623 | -0.6048 | Yes |
| 175 | NCAPD2 |  |  | 2089 | -0.627 | -0.6026 | Yes |
| 176 | HNRNPU |  |  | 2090 | -0.628 | -0.5994 | Yes |
| 177 | MCM8 |  |  | 2092 | -0.635 | -0.5966 | Yes |
| 178 | KIF18A |  |  | 2093 | -0.636 | -0.5934 | Yes |
| 179 | FAM111B |  |  | 2094 | -0.637 | -0.5901 | Yes |
| 180 | MIS18A |  |  | 2096 | -0.639 | -0.5873 | Yes |
| 181 | RFC3 |  |  | 2098 | -0.642 | -0.5845 | Yes |
| 182 | HMGN1 |  |  | 2099 | -0.646 | -0.5812 | Yes |
| 183 | BRCA2 |  |  | 2100 | -0.650 | -0.5779 | Yes |
| 184 | ITGB3BP |  |  | 2102 | -0.652 | -0.5750 | Yes |
| 185 | NCAPH |  |  | 2104 | -0.652 | -0.5722 | Yes |
| 186 | CKAP2L |  |  | 2109 | -0.663 | -0.5708 | Yes |
| 187 | ALYREF |  |  | 2111 | -0.677 | -0.5678 | Yes |
| 188 | LSM3 |  |  | 2114 | -0.694 | -0.5653 | Yes |
| 189 | CKS2 |  |  | 2115 | -0.694 | -0.5617 | Yes |
| 190 | CDC20 |  |  | 2117 | -0.695 | -0.5587 | Yes |
| 191 | RAD21 |  |  | 2118 | -0.697 | -0.5551 | Yes |
| 192 | ANP32B |  |  | 2119 | -0.700 | -0.5515 | Yes |
| 193 | CDCA2 |  |  | 2120 | -0.705 | -0.5479 | Yes |
| 194 | HDGF |  |  | 2124 | -0.709 | -0.5457 | Yes |
| 195 | CENPM |  |  | 2125 | -0.709 | -0.5421 | Yes |
| 196 | HNRNPD |  |  | 2126 | -0.713 | -0.5384 | Yes |
| 197 | RAD51AP1 |  |  | 2127 | -0.715 | -0.5348 | Yes |
| 198 | CDCA8 |  |  | 2129 | -0.722 | -0.5316 | Yes |
| 199 | CKAP2 |  |  | 2131 | -0.729 | -0.5283 | Yes |
| 200 | ATAD5 |  |  | 2133 | -0.742 | -0.5250 | Yes |
| 201 | VRK1 |  |  | 2134 | -0.743 | -0.5212 | Yes |
| 202 | KIF2C |  |  | 2136 | -0.756 | -0.5178 | Yes |
| 203 | BRCA1 |  |  | 2138 | -0.758 | -0.5144 | Yes |
| 204 | KIF14 |  |  | 2141 | -0.773 | -0.5115 | Yes |
| 205 | NDC80 |  |  | 2142 | -0.775 | -0.5075 | Yes |
| 206 | H2AFV |  |  | 2143 | -0.779 | -0.5035 | Yes |
| 207 | FBXO5 |  |  | 2144 | -0.779 | -0.4995 | Yes |
| 208 | KIF23 |  |  | 2145 | -0.782 | -0.4954 | Yes |
| 209 | GMNN |  |  | 2146 | -0.785 | -0.4914 | Yes |
| 210 | MELK |  |  | 2148 | -0.786 | -0.4879 | Yes |
| 211 | ANLN |  |  | 2149 | -0.797 | -0.4838 | Yes |
| 212 | SMC3 |  |  | 2150 | -0.800 | -0.4797 | Yes |
| 213 | KIFC1 |  |  | 2151 | -0.802 | -0.4755 | Yes |
| 214 | DEPDC1 |  |  | 2152 | -0.809 | -0.4714 | Yes |
| 215 | HIST1H2BH |  |  | 2153 | -0.811 | -0.4672 | Yes |
| 216 | DEK |  |  | 2154 | -0.817 | -0.4630 | Yes |
| 217 | DUT |  |  | 2155 | -0.824 | -0.4588 | Yes |
| 218 | NCAPG2 |  |  | 2156 | -0.827 | -0.4545 | Yes |
| 219 | TUBB |  |  | 2157 | -0.831 | -0.4502 | Yes |
| 220 | NUCKS1 |  |  | 2158 | -0.832 | -0.4460 | Yes |
| 221 | MCM7 |  |  | 2160 | -0.839 | -0.4422 | Yes |
| 222 | TUBB4B |  |  | 2162 | -0.843 | -0.4383 | Yes |
| 223 | ECT2 |  |  | 2163 | -0.846 | -0.4340 | Yes |
| 224 | HMGB3 |  |  | 2164 | -0.852 | -0.4296 | Yes |
| 225 | KIF20B |  |  | 2165 | -0.852 | -0.4252 | Yes |
| 226 | SPC24 |  |  | 2166 | -0.853 | -0.4208 | Yes |
| 227 | ARHGAP11A |  |  | 2167 | -0.855 | -0.4164 | Yes |
| 228 | DTYMK |  |  | 2168 | -0.858 | -0.4120 | Yes |
| 229 | MCM10 |  |  | 2169 | -0.858 | -0.4076 | Yes |
| 230 | BUB1 |  |  | 2170 | -0.860 | -0.4032 | Yes |
| 231 | FOXM1 |  |  | 2171 | -0.861 | -0.3988 | Yes |
| 232 | CDCA5 |  |  | 2172 | -0.866 | -0.3943 | Yes |
| 233 | LBR |  |  | 2173 | -0.870 | -0.3898 | Yes |
| 234 | BUB1B |  |  | 2174 | -0.871 | -0.3853 | Yes |
| 235 | NUF2 |  |  | 2176 | -0.877 | -0.3813 | Yes |
| 236 | KPNA2 |  |  | 2178 | -0.894 | -0.3773 | Yes |
| 237 | CCNA2 |  |  | 2181 | -0.902 | -0.3736 | Yes |
| 238 | CHAF1A |  |  | 2182 | -0.903 | -0.3690 | Yes |
| 239 | MIS18BP1 |  |  | 2183 | -0.903 | -0.3644 | Yes |
| 240 | KIF15 |  |  | 2185 | -0.915 | -0.3602 | Yes |
| 241 | KIF4A |  |  | 2186 | -0.921 | -0.3554 | Yes |
| 242 | PRR11 |  |  | 2187 | -0.930 | -0.3507 | Yes |
| 243 | ORC6 |  |  | 2188 | -0.937 | -0.3458 | Yes |
| 244 | C21orf58 |  |  | 2189 | -0.937 | -0.3410 | Yes |
| 245 | CENPW |  |  | 2192 | -0.943 | -0.3372 | Yes |
| 246 | TUBA1B |  |  | 2193 | -0.947 | -0.3323 | Yes |
| 247 | LMNB1 |  |  | 2194 | -0.950 | -0.3274 | Yes |
| 248 | HJURP |  |  | 2195 | -0.962 | -0.3225 | Yes |
| 249 | UBE2T |  |  | 2196 | -0.965 | -0.3175 | Yes |
| 250 | FANCI |  |  | 2198 | -0.966 | -0.3131 | Yes |
| 251 | CDKN3 |  |  | 2199 | -0.979 | -0.3080 | Yes |
| 252 | CCNB2 |  |  | 2200 | -0.983 | -0.3030 | Yes |
| 253 | CKS1B |  |  | 2201 | -0.987 | -0.2979 | Yes |
| 254 | HIST1H1E |  |  | 2202 | -0.992 | -0.2928 | Yes |
| 255 | ESCO2 |  |  | 2204 | -1.004 | -0.2881 | Yes |
| 256 | SMC2 |  |  | 2205 | -1.006 | -0.2830 | Yes |
| 257 | KIF11 |  |  | 2206 | -1.008 | -0.2778 | Yes |
| 258 | EZH2 |  |  | 2207 | -1.017 | -0.2725 | Yes |
| 259 | SPC25 |  |  | 2208 | -1.019 | -0.2673 | Yes |
| 260 | XRCC2 |  |  | 2210 | -1.028 | -0.2625 | Yes |
| 261 | CENPE |  |  | 2211 | -1.036 | -0.2572 | Yes |
| 262 | DHFR |  |  | 2212 | -1.040 | -0.2518 | Yes |
| 263 | NUDT1 |  |  | 2214 | -1.054 | -0.2469 | Yes |
| 264 | MYBL2 |  |  | 2215 | -1.058 | -0.2415 | Yes |
| 265 | CLSPN |  |  | 2216 | -1.060 | -0.2360 | Yes |
| 266 | CENPN |  |  | 2217 | -1.079 | -0.2305 | Yes |
| 267 | UBE2C |  |  | 2218 | -1.081 | -0.2249 | Yes |
| 268 | CENPU |  |  | 2219 | -1.088 | -0.2193 | Yes |
| 269 | H2AFZ |  |  | 2220 | -1.090 | -0.2137 | Yes |
| 270 | HMGB1 |  |  | 2221 | -1.116 | -0.2080 | Yes |
| 271 | NCAPG |  |  | 2222 | -1.122 | -0.2022 | Yes |
| 272 | CCNB1 |  |  | 2224 | -1.148 | -0.1968 | Yes |
| 273 | CDK1 |  |  | 2225 | -1.153 | -0.1909 | Yes |
| 274 | PTTG1 |  |  | 2226 | -1.198 | -0.1847 | Yes |
| 275 | ANP32E |  |  | 2227 | -1.200 | -0.1786 | Yes |
| 276 | MAD2L1 |  |  | 2228 | -1.204 | -0.1724 | Yes |
| 277 | GTSE1 |  |  | 2230 | -1.220 | -0.1666 | Yes |
| 278 | DBF4 |  |  | 2232 | -1.247 | -0.1607 | Yes |
| 279 | HMGN2 |  |  | 2233 | -1.267 | -0.1542 | Yes |
| 280 | RRM2 |  |  | 2234 | -1.268 | -0.1477 | Yes |
| 281 | DLGAP5 |  |  | 2235 | -1.273 | -0.1411 | Yes |
| 282 | SMC4 |  |  | 2236 | -1.281 | -0.1345 | Yes |
| 283 | PBK |  |  | 2237 | -1.325 | -0.1277 | Yes |
| 284 | ATAD2 |  |  | 2238 | -1.381 | -0.1206 | Yes |
| 285 | TMPO |  |  | 2239 | -1.382 | -0.1135 | Yes |
| 286 | PRC1 |  |  | 2240 | -1.397 | -0.1063 | Yes |
| 287 | HMGB2 |  |  | 2241 | -1.469 | -0.0987 | Yes |
| 288 | BIRC5 |  |  | 2242 | -1.469 | -0.0912 | Yes |
| 289 | HIST1H1C |  |  | 2243 | -1.485 | -0.0836 | Yes |
| 290 | ASPM |  |  | 2244 | -1.490 | -0.0759 | Yes |
| 291 | TOP2A |  |  | 2246 | -1.562 | -0.0684 | Yes |
| 292 | NUSAP1 |  |  | 2247 | -1.600 | -0.0601 | Yes |
| 293 | TPX2 |  |  | 2248 | -1.609 | -0.0519 | Yes |
| 294 | CENPF |  |  | 2249 | -1.691 | -0.0432 | Yes |
| 295 | HIST1H1D |  |  | 2250 | -1.720 | -0.0343 | Yes |
| 296 | HIST1H1B |  |  | 2251 | -1.796 | -0.0251 | Yes |
| 297 | MKI67 |  |  | 2252 | -1.892 | -0.0153 | Yes |
| 298 | HIST1H4C |  |  | 2253 | -2.984 | 0.0000 | Yes |
Table: GSEA details [plain text format]

  

Fig 2: RICHARDS\_NOWAKOWSKI\_SCIENCE\_2017\_MGE\_IPC2\_UPREG: Random ES distribution      
 Gene set null distribution of ES for **RICHARDS\_NOWAKOWSKI\_SCIENCE\_2017\_MGE\_IPC2\_UPREG**
